# Exploring ultrasound-induced metabolic attenuation in *Lacticaseibacillus casei* ATCC 393—A combined approach using traditional methods and flow cytometry

**DOI:** 10.3389/fmicb.2025.1589054

**Published:** 2025-06-26

**Authors:** Irene Giordano, Mohammed Salman, Stefania Arioli, Diego Mora, Gianluigi Mauriello

**Affiliations:** ^1^Department of Agricultural Sciences, University of Naples Federico II, Naples, Italy; ^2^Department of Food Environmental and Nutritional Sciences (DeFENS), University of Milan, Milan, Italy

**Keywords:** probiotic, ultrasound, attenuation, membrane integrity, metabolic activity

## Abstract

Attenuation technologies applied to probiotics aim to modulate specific metabolic pathways, particularly acidification, while maintaining cell viability. Although ultrasound is an emerging tool in this context, its precise mechanism of action on probiotic cells remains poorly understood. The study aimed to establish a suitable method to investigate the effects of ultrasound attenuation on probiotics. *Lacticaseibacillus casei* ATCC 393 was exposed to sonication for 6 and 8 min in a water suspension. Morphological changes, cultivability, acidification capacity, and growth recovery were assessed using culture-dependent methods. Flow cytometry (FCM) combined with fluorescent staining was used to evaluate membrane integrity (as a marker of viability) and esterase activity (as a marker of metabolic activity). Moreover, plate count and FCM data were compared to estimate the overall effect of ultrasound. A reduction in cell size was observed, which was confirmed by decreases in forward and side scatter signals. Acidification capacity was dependent on the intensity applied, and only the 8-min treatment induced prolonged modulation over 24 h. Esterase activity was similarly affected by both sonicating times, whereas membrane integrity reduction was dependent on the treatment intensity. The probiotic demonstrated the ability to restore growth, with recovery time proportionally increasing with the duration of ultrasound treatment. Direct comparisons of the viable, culturable, and metabolically active subpopulations indicate that they are similarly affected by 6 min of sonication. On the contrary, 8 min of sonication increased sample heterogeneity, generating three different subpopulations. The lack of overlap between viable and culturable clusters suggested that the cells that were sonicated for 8 min entered the viable but non-culturable state. These results provide insight into the intensity-dependent effects of ultrasound on probiotic functionality and demonstrate the value of integrative analytical approaches (FCM combined with traditional methods) for characterizing bacterial responses to attenuation strategies.

## 1 Introduction

In the modern era, unconventional technologies have taken a central role in the food industry. Among other technologies, ultrasound treatment stands out as a green, eco-friendly, energy-efficient, time-saving, and cost-effective solution (Khaire et al., 2022; Mgoma et al., 2025). Ultrasounds are sound waves with frequencies above 20 kHz and up to 2 MHz that generate cavitation bubbles as they pass through a liquid medium. Mechanical and chemical events (temperature and pressure increase, high shear forces, and free radical generation) occur because of the implosion of the cavitation bubbles. These events can be helpful for several purposes in the food field: (i) oil and bioactive molecules extraction (Clodoveo, 2019; Ferrentino et al., 2020; Kumar et al., 2021; Thilakarathna et al., 2023); (ii) enzyme inactivation or enhancement (Vanga et al., 2021); (iii) emulsification (Yao et al., 2022); (iv) freezing and crystallization (Yao et al., 2022); (v) food preservation (Khaire et al., 2022); and (vi) microbial metabolism modulation (Bevilacqua et al., 2016; Racioppo et al., 2017; Giordano and Mauriello, 2023).

Although microbial inactivation by ultrasound has been extensively studied, only a few studies in the literature (Bevilacqua et al., 2016; Racioppo et al., 2017; Giordano and Mauriello, 2023) investigated the potential of ultrasound in modulating the metabolism of probiotic cells. While the term modulation refers to both enhancement and reduction of some metabolic pathways, attenuation specifically refers to strategies aimed at reducing or weakening certain metabolic pathways.

Ultrasounds interact with bacterial cells at multiple levels. This multiparametric technique offers flexibility for targeting specific cellular processes. However, the complexity of the phenomena triggered in bacteria represents a major limitation in terms of predictability and standardization. Indeed, it has been demonstrated that the phenotypical alteration observed in sonicated cells is correlated with a genetic alteration at the transcriptional level (Ojha et al., 2018; Giordano et al., 2024). Therefore, knowing the target of ultrasound on bacteria and identifying the specific cellular targets enables the employment of ultrasound to specifically attenuate certain microbial activity.

Ultrasound attenuation of the sugar metabolism in probiotic bacteria may represent an innovative approach to formulating functional foods, enabling the preservation of the product's intrinsic characteristics. Once added to a food matrix, these attenuated probiotics are unable to metabolize nutrients and release metabolites, such as organic acids. Therefore, an understanding of the mechanisms by which attenuation occurs could support the expansion of the probiotic food market by offering consumer products with improved acceptability.

Attenuation has two basic requirements: modulation of probiotic performances and retention of cell viability. Although the terms cultivability and viability have been traditionally used as synonymous, they refer to two distinct properties. Cultivability refers to the ability of a cell to reproduce or form a colony on solid media. A truly viable cell is cultivable, metabolically active, and structurally intact (Davis, 2014). However, after exposure to a stressful environment or treatment, such as ultrasound, the cells can exhibit a different degree of response, resulting in the generation of subpopulations in various physiological states. These subpopulations could be viable, viable but nonculturable (VBNC), dormant, or dead. VBNC cells lost their cultivability but retain some metabolic activity. Dormant cells, in contrast, lack cultivability and metabolic activity but retain an intact membrane (Trinh and Lee, 2022). Thus, plate counts are not suitable for detecting unculturable cell clusters generated during ultrasound treatment. This leads to the underestimation of the real effect of the applied therapy on microbial viability and prevents the detection of other subpopulations. Therefore, a multiparametric analysis is needed to assess the several effects of ultrasound treatment on microbial cells. Flow cytometry (FCM) offers an advanced method for overcoming the limitations of traditional approaches by enabling the detection and discrimination of subpopulations that share structural and physiological properties within a cell culture or microbial community. In FCM analysis, initial discrimination is made based on the phenomenon of light scattering. At the same time, further distinction is achieved by staining the cells with two or more dyes that emit fluorescent light when exposed to a light beam (Wilkinson, 2018). The physiological status of individual cells within a heterogeneous population can be characterized using fluorescent probes that target specific cellular functions, such as membrane integrity and enzymatic activity. A combination of membrane-permeant dyes (e.g., the SYTO family) and membrane-impermeant nucleic acid-binding dyes (e.g., propidium iodide, PI) is commonly used to assess membrane integrity. Metabolic activity is often evaluated based on esterase function, as esterases represent a broad class of enzymes capable of cleaving ester bonds regardless of the specific substrate. A widely used probe is carboxyfluorescein diacetate (cFDA), a non-fluorescent compound that is converted to fluorescent carboxyfluorescein (cF) by intracellular esterases in metabolically active cells (Zand et al., 2021).

This study aimed to evaluate the feasibility of combining traditional methods with flow cytometry to provide a comprehensive assessment of ultrasound-induced attenuation effects on a model probiotic. Specifically, this study investigated the use of ultrasound to modulate the sugar metabolism of the probiotic *Lacticaseibacillus casei* ATCC 393 (*L. casei* ATCC 393). The research also focused on cell morphology characterized by light microscope observation and light scattering detection. Acidification ability and esterase activity, measured by flow cytometry, were used to evaluate the metabolic state of the probiotic after ultrasound attenuation. Finally, a growth curve was constructed to assess the probiotic's ability to restore its normal physiology after sonication. Overall, the findings offer valuable insights into the application of ultrasound for modulating probiotic metabolism.

## 2 Materials and methods

### 2.1 Probiotic culture

The strain *L. casei* ATCC 393 was used as the probiotic culture (Hill et al., 2018) due to its widespread use in food applications (Abdel-Hamid et al., 2019) and its well-documented beneficial effects after administration (Aindelis et al., 2020; Qiao et al., 2022). The strain was grown in deMan, Rogosa, and Sharpe (MRS) broth (OXOID Ltd., Basingstoke, Hampshire, UK) for 18 h at 37°C. A volume of 30 ml was used for the sonication experiment. The pellet was collected by centrifugation (6,000 *g*, 10 min) (ALC^®^ Centrifuge PK130, Thermo Fisher Scientific, Waltham, MA, USA) and suspended in an equal volume of sterile deionized water at a final cell density of 9 Log CFU/mL (Racioppo et al., 2017).

### 2.2 Sonication experiment

Ultrasound treatment was carried out as previously described by Giordano and Mauriello (2023). Briefly, LABSONIC U (B. Braun) was set at a 50% duty cycle (DC) in pulsing mode for sonication times of 6 and 8 min. The operating frequency was 20 kHz; the power was set at 57 W, and the mean efficiency ranged from 77 to 84%. Prior to use, the probe was cleaned with 70% ethanol. To prevent an increase in cell suspension temperature, the sample was kept on ice both during and immediately after sonication treatment.

### 2.3 Flow cytometry

Sonicated and non-sonicated cell suspensions were properly diluted in phosphate buffered saline (PBS) (NaCl 0.15 M, KH_2_PO_4_ 1 mM, Na_2_HPO_4_ 3 mM, pH 7.4), previously sterilized (121°C, 15 min) and filtered (0.22 μm) immediately prior to use to avoid noise interference with sample reading. A volume of 50 μL per sample was analyzed with a C6^®^ Plus BD Accuri flow cytometer (BD Biosciences, Milan, Italy) at a medium flow rate. The FCM analysis was performed using thresholds of 3,000 for Forward Scatter Height (FSC-H) and 1,000 for Side Scatter Height (SSC-H). All parameters were collected as logarithmic signals. Esterase activity and viability of *L. casei* in both pre-treatment and post-treatment samples were evaluated according to protocols A and B, respectively, of the International Organization for Standardization (ISO) 19344 (ISO, 2015).

#### 2.3.1 Viability assay

Cell viability and membrane integrity were evaluated by simultaneously staining with SYTO 24™ and propidium iodide (PI), which are cell-permeant and impermeant nucleic acid dyes, respectively (Arioli et al., 2022). The staining reaction was carried out in a 1 ml final volume, with SYTO 24™ 0.1 μM and PI 0.2 μM. Samples were gently mixed and incubated in the dark for 15 min at 37°C.

#### 2.3.2 Esterase activity assay

Intracellular esterase activity was detected through a double staining with carboxyfluorescein diacetate (cFDA) and PI. In metabolically active cells, cFDA, a non-fluorescent molecule, is converted by esterase into carboxyfluorescein (cF, excitation 488 nm, emission 620 nm), an impermeant green fluorescent dye. The staining procedure was done in two steps: Briefly, 10 μL of cFDA (5 mM) was added to 970 μL of cell suspension in PBS and incubated at 30°C for 15 min in the dark. Then, 20 μL of PI (1.5 mM) (excitation 488 nm, emission 630 nm) were added to the mixture, gently mixed, and incubated in the dark for 15 min at room temperature (ISO 19344) (ISO, 2015).

#### 2.3.3 Morphological evaluation

Light scattering was used to evaluate morphological changes after sonication. Specifically, FSC (dimension) and SSC (internal complexity) were compared pre-treatment and post-treatment for each sample. Data were reported as percentage reduction.

#### 2.3.4 Flow cytometry data analysis

The fluorescent signals were collected in a density plot, SYTO 24™ or cF vs. PI. A green signal was detected from live and metabolically active cells with an intact membrane impermeable to PI. Both green and red signals were detected from damaged cells with membrane permeable to both dyes and reduced metabolic activity. A red signal was detected from dead cells with a permeabilized membrane unable to retain SYTO 24™ and unable to metabolize cFDA. To properly identify subpopulations, color compensation was performed, and distinct cell groups were obtained.

### 2.4 Ultrasound treatment effect on acidification and growth rates

Attenuation was evaluated in terms of acidification capacities by inoculating sonicated and non-sonicated cells (1% v/v) in MRS broth. Samples were incubated at 37°C, and the pH was monitored after 6 and 24 h (Racioppo et al., 2017). Data were reported as ΔpH.

Serial decimal dilutions in quarter-strength Ringer solution (OXOID Ltd., Basingstoke, Hampshire, UK) and spread plate count on MRS agar were carried out to evaluate cell cultivability. The plates were then incubated at 37°C for 48 h in anaerobic conditions.

To test the *L. casei* ATCC 393's ability to restore its metabolism, MRS broth was inoculated at 0.6 ± 0.1 OD_600_ immediately after the sonication experiment with sonicated cells and disposed of in triplicate in a 96-well microtiter plate. MRS broth inoculated with non-sonicated cells was used as a positive control, and sterile MRS broth was used as a negative control. The plate was incubated at 37°C in a microplate reader (Eon Microplate Spectrophotometer, BioTek Instruments, Inc., CA, USA) for 48 h. The OD_600_ was read every 15 min. Growth parameters were analyzed using Gen5 software (AGILENT, Santa Clara, CA, United States) as the mean of three independent determinations. Data were then plotted on an OD_600_ vs. time (h) graph. The incubation time needed to reach the same cell physiology stage (early stationary phase growth) was also determined by applying Equation 1 (Ferrando et al., 2015) to the data modeled according to the modified Gompertz equation (Zwietering et al., 1990):


(1) $$\begin{array}{l} \text{Maximum specif speed }\left(\mu_{\max}\right)=\frac{{\text{lnOD}}_{f}-{\text{lnOD}}_{i}}{t_{f}-t_{i}}, \end{array}$$

where OD_f_ is the optical density measured at the early stationary phase; OD_i_ is the initial optical density; *t*_f_ is the time of the early stationary phase; *t*_i_ is the initial time.

### 2.5 Statistical analysis

Three independent replicates were performed for each experiment. The collected data were then analyzed using the Statistical Package for the Social Sciences (SPSS, IBM Statistics) software, employing a one-way analysis of variance (ANOVA) and paired or unpaired Student's *t*-tests. The results were reported as mean value (*n* = 3) ± the standard deviation (SD), and the significance was declared at a *p*-value of < 0.05.

## 3 Results

### 3.1 Cell morphology

**Figure 1:**
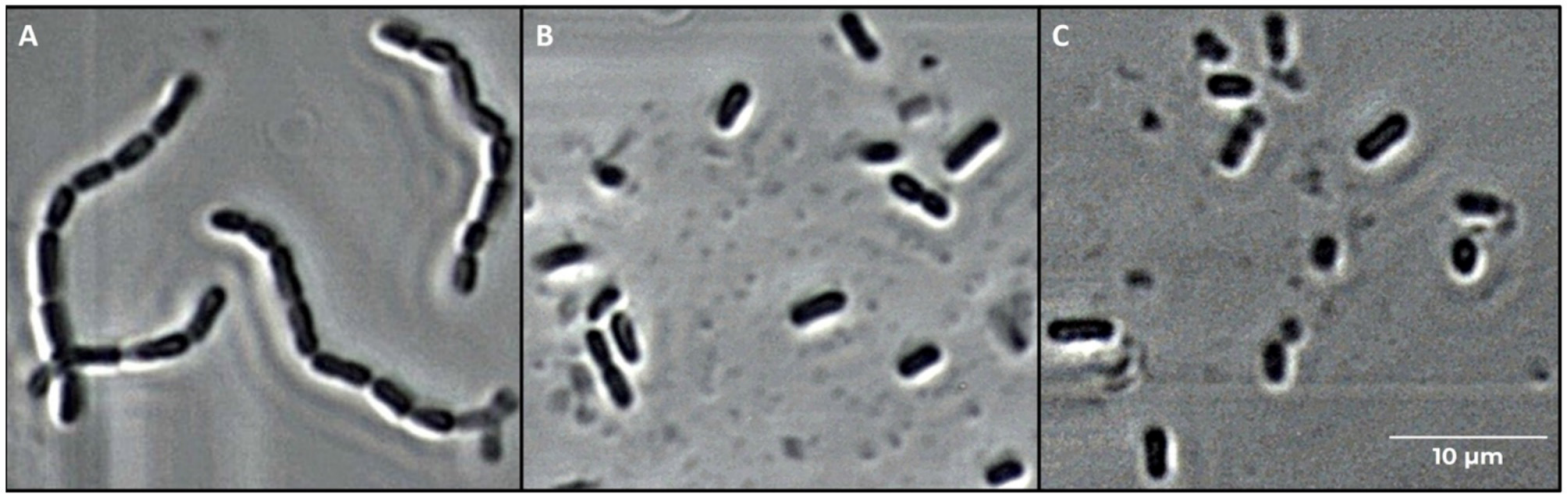
Microscope images (400 × magnification) of *Lacticaseibacillus casei* ATCC 393 cells. **(A)** Untreated, **(B)** sonicated for 6 min, and **(C)** sonicated for 8 min.

**Figure 2:**
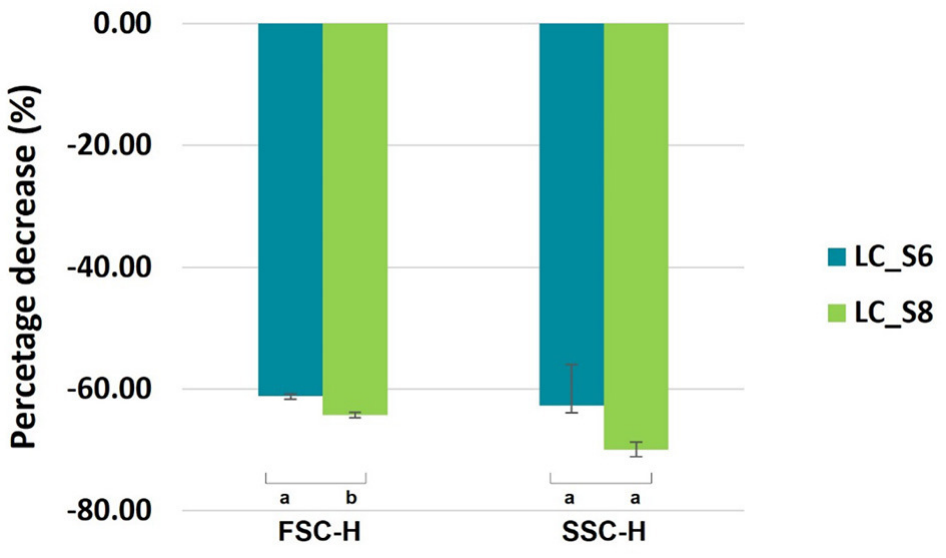
Percentage decrease (mean values ± SD, *n* = 3) Forward Scatter Height (FSC-H) and Side Scatter Height (SSC-H) angles of *Lacticaseibacillus casei* ATCC 393 detected by FCM after ultrasound treatment at 6 and 8 min. LC_S6: *L. casei* sonicated for 6 min and LC_S8: *L. casei* sonicated for 8 min. Different letters indicate a significant difference at a *p*-value of <0.05.

**Figure 3:**
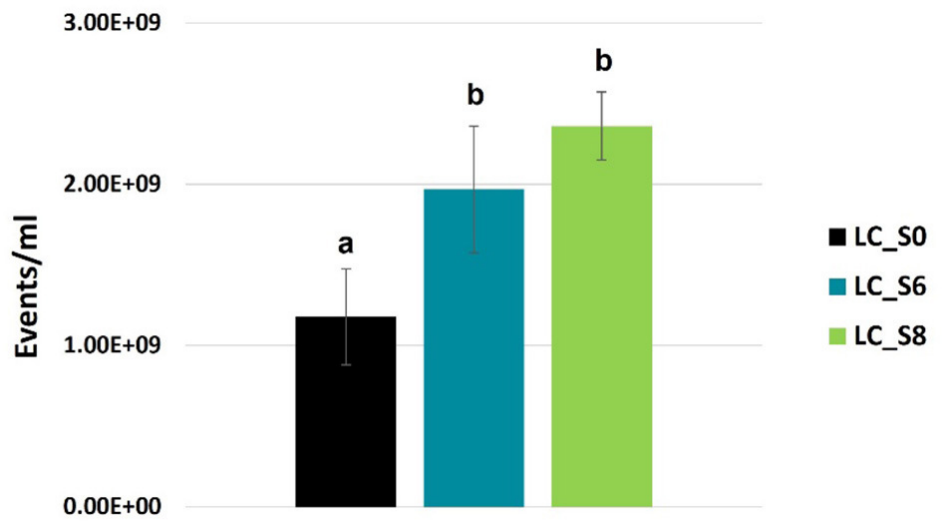
FCM-based quantification of total events (events/mL, mean values ± SD, *n* = 3) of *Lacticaseibacillus casei* ATCC 393 water suspension. LC_S0: not sonicated, control; LC_S6: 6 min sonicated *L. casei*; LC_S8: and 8 min sonicated *L. casei*. Different letters indicate a significant difference at *p* < 0.05.

Phase contrast microscope images of *L. casei* ATCC 393 before and after sonication are shown in Figure 1. As with other bacteria of the species *Lacticaseibacillus casei*, it is characterized by a growth in chains and a streptococcal morphology with a typical curvature. Sonicated bacteria exhibited a single-cell morphology, resulting in a smaller, rod-shaped cell compared to the control bacteria. Morphology variation was also detected by FCM analysis (Figure 2). A reduction in Forward Scatter C (FSC) and Side Scatter C (SSC) parameters was recorded for both sonicated samples compared to the control sample. However, a significant (*p* < 0.05) and a non-significant time-related effect were observed for the percentage decrease in FSC and SSC, respectively. The change toward a single-cell morphology was also confirmed by an increase in the total number of collected events (Figure 3).

### 3.2 Cell viability and membrane integrity assessment by FCM

**Figure 4:**
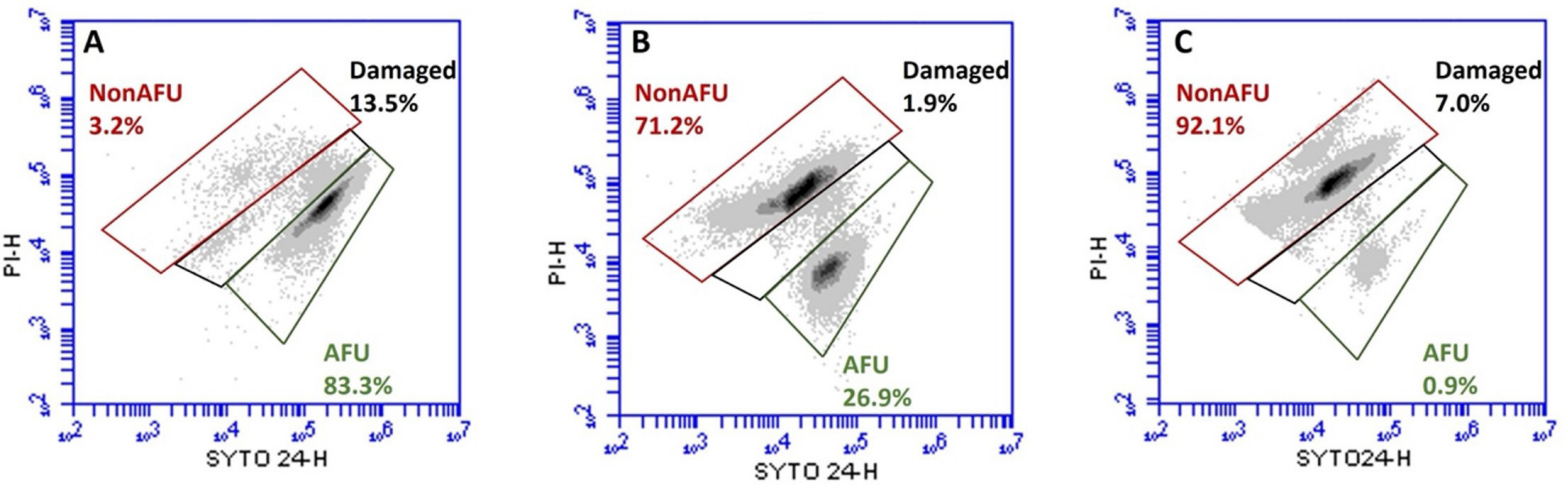
Representative flow cytometry density plot of SYTO 24™ vs. PI of *Lacticaseibacillus casei* ATCC 393 before **(A)** and after ultrasound treatment for 6 **(B)** and 8 **(C)** min. Subpopulations detected: Active Fluorescent Unit (AFU), viable cells (SYTO^+^ and PI^−^); Damaged (sublethally damaged cells, SYTO^+^ and PI^+^); Non-Active Fluorescent Unit (Non-AFU), dead cells (SYTO^−^ and PI^+^).

FCM analyses allowed us to selectively count three subpopulations based on fluorescence: (i) the green population, consisting of SYTO 24™ positive cells, defined as Active Fluorescent Units (AFU) and considered live cells; (ii) the green and red population, comprising SYTO 24™ and PI double-positive cells, labeled as damaged; (iii) the red population, consisting of PI positive cells, defined as Non-Active Fluorescent Units (Non-AFU) and considered dead cells. FCM viability data are reported in Figure 4 as the percentage of subpopulations.

Figure 4A shows that the majority of the population in the untreated sample (LC_S0) is represented by live cells (83.3%). Figure 4B shows that 26.9% of the sampling population sonicated for 6 min (LC_S6) have an intact membrane, while 71.2% have a permeabilized one. Finally, Figure 4C shows the reduction of the *L. casei* ATCC 393 subpopulation with an intact membrane to 0.9% and the dramatic increase of the subpopulation with a permeabilized membrane to 92.1% after 8 min of sonication (LC_S8).

The FCM viability data were further converted to the number of detected events (Log events/ml) and summarized in Table 1.

**Table 1:** Quantification of subpopulations of *Lacticaseibacillus casei* ATCC 393 (Log/mL of events, mean values ± SD, *n* = 3) detected by FCM analysis through SYTO 24™ and PI double staining.

**Sample**	**AFU**	**Damaged**	**Non-AFU**
LC_S0	8.88 ± 0.03^a^	7.92 ± 0.58^a^	7.38 ± 0.36^a^
LC_S6	8.60 ± 0.08^b^	7.45 ± 0.08^a^	9.02 ± 0.11^b^
LC_S8	7.21 ± 0.16^c^	7.84 ± 0.85^a^	9.26 ± 0.06^b^

LC_S0, non-sonicated, control; LC_S6, 6 min treatment; LC_S8, 8 min treatment.

Active Fluorescent Unit (AFU): viable cells, SYTO^+^ and PI^−^; Damaged: sublethally damaged cells, SYTO^+^ and PI^+^; Non-Active Fluorescent Unit (Non-AFU): dead cells, SYTO^−^, and PI^+^.

Different letters in the same column indicate that the differences between the samples are significant (*p* < 0.05).

Sonication treatments significantly (*p* < 0.05) decreased the AFU subpopulation in a time-dependent manner. In the control sample (LC_S0), the AFU subpopulation was 8.88 Log events/mL, which was reduced to 8.60 and 7.21 Log events/mL after 6 (LC_S6) and 8 (LC_S8) min of treatment, respectively. Moreover, the damaged subpopulation remained constant across all samples with no significant differences (*p* > 0.05) between the control and sonicated samples. Finally, a considerable increase (*p* < 0.05) in the Non-AFU subpopulation was observed in both sonicated samples. However, no significant differences were found between the two sonicated samples for the Non-AFU subpopulation (*p* > 0.05).

### 3.3 Metabolic activity

*L. casei* ATCC 393 metabolic activity after sonication was evaluated through the assessment of the acidification rate and esterase activity. These two measures were used to gauge the probiotic's metabolic response following ultrasound treatment, providing insights into how sonication affected its ability to acidify the medium and perform essential metabolic functions.

#### 3.3.1 Acidification activity

Data of ΔpH are shown in Table 2.

**Table 2:** Decrease of pH (mean values ± SD, *n* = 3) of MRS broth inoculated with cells of sonicated and non-sonicated *Lacticaseibacillus casei* ATCC 393, after 6 and 24 h of incubation at 37°C.

**Sample**	**Time of incubation (h)**	
	**6**	**24**
LC_S0	0.57 ± 0.02^a^	2.26 ± 0.02^a^
LC_S6	0.21 ± 0.01^b^	2.05 ± 0.02^a^
LC_S8	0.12 ± 0.02^c^	0.78 ± 0.15^b^

LC_S0, non-sonicated cells, control; LC_S6, cells sonicated for 6 min; LC_S8, cells sonicated for 8 min.

Different letters in the same column indicate significant differences (*p* < 0.05).

The 6-min treatment induced a transient attenuation in the acidification ability of *L. casei* ATCC 393. In fact, after 6 h of incubation, the acidification caused by the LC_S6 was significantly (*p* < 0.05) lower than that of the control (0.21 and 0.57, respectively). However, the probiotic restored its acidification capabilities after 24 h of incubation with a ΔpH of ~2.0 for both LC_S0 (control) and LC_S6 (6 min sonicated sample). Instead, the LC_S8 (8 min sonicated sample) exhibited a significantly (*p* < 0.05) lower acidification at both time points, with a ΔpH of 0.12 at *t*_6_ and 0.78 at *t*_24_.

#### 3.3.2 Esterase activity

**Figure 5:**
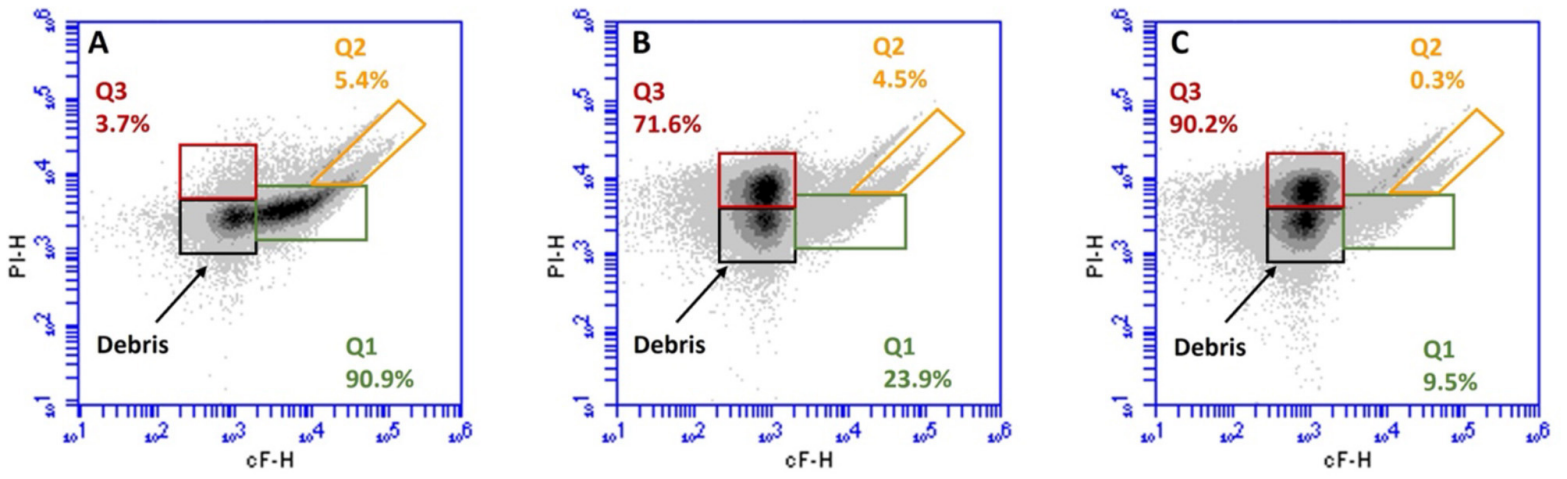
Representative flow cytometry density plot of cF vs. PI of *Lacticaseibacillus casei* ATCC 393 before **(A)** and after 6 **(B)**, and 8 **(C)** min of sonication. Subpopulations detected: Q1, metabolically active cells (cF^+^ and PI^−^); Q2, partially active cells (cF^+^ and PI^+^); Q3, non-metabolically active cells (cF^−^ and PI^+^); and debris: unmarked cells (cF^−^ and PI^−^).

Simultaneous staining with cFDA and PI allowed us to distinguish four main subpopulations, according to Protocol A of ISO 19344 (ISO, 2015; Figure 5): (i) cells with undamaged membranes and active esterase activity were labeled as Q1 (cF^+^ and PI^−^, green signal), which were considered as metabolically active cells; (ii) cells with damaged membranes and residual esterase activity were labeled as Q2 (cF^+^ and PI^+^, green and red signal), which were considered as partially metabolically active cells; (iii) cells with permeabilized membranes and inactive esterase were labeled as Q3 (cF^−^ and PI^+^, red signal), which were considered as non-metabolically active cells; and (iv) unstained debris were excluded from the calculation of subpopulation percentage (cF^−^ and PI^−^, no fluorescent signal).

Figure 5 shows the distribution of subpopulations Q1, Q2, and Q3 in *L. casei* ATCC 393 before and after ultrasound treatment. According to the viability test, in the untreated sample, 90.9% of the population (Q1) was metabolically active (cF^+^) with an intact membrane (PI^−^; Figure 5A). However, it was reduced by the ultrasound treatments, which generated a varying metabolic response depending on the duration of the treatment. The subpopulation Q1 represents 23.9% of the total collected events in sample LC_S6 (Figure 5B), while it accounted for 9.5% of the total collected events in sample LC_S8 (Figure 5C). Moreover, after 6 min of sonication (Figure 5B), the partially metabolically active (cF^+^) and sublethally injured (PI^+^) subpopulation (Q2) remained stable at 4.5%. In contrast, the Q2 subpopulation dropped to just 0.3% after 8 min of sonication (panel C). Finally, an increase in non-metabolically active (cF^−^) with a damaged membrane (PI^+^) subpopulation (Q3) was observed in both LC_S6 and LC_S8. The Q3 subpopulation increased to 71.6 and 90.2% after 6 and 8 min of ultrasound treatment, respectively.

The FCM-detected events were also converted into Log events/mL and reported in Table 3.

**Table 3:** Quantification of subpopulations of *Lacticaseibacillus casei* ATCC 393 (Log/mL of events, mean values ± SD, *n* = 3) detected by FCM analysis through cFDA and PI double staining.

**Sample**	**Q1**	**Q2**	**Q3**
LC_S0	8.96 ± 0.18^a^	7.73 ± 0.24^a^	7.55 ± 0.10^a^
LC_S6	8.58 ± 0.05^b^	7.86 ± 0.14^a^	9.07 ± 0.10^b^
LC_S8	8.37 ± 0.01^b^	6.94 ± 4.01^a^	9.35 ± 0.04^b^

LC_S0, non-sonicated, control; LC_S6, 6 min treatment; LC_S8, 8 min treatment.

Subpopulations detected: Q1, metabolically active cells (cF^+^ and PI^−^); Q2, partially active cells (cF^+^ and PI^+^); Q3, non-metabolically active cells (cF^−^ and PI^+^).

Different letters in the same column indicate significant differences (*p* < 0.05).

As also observed in Figure 3, the metabolically active subpopulation Q1 (cF^+^ and PI^−^) was reduced by both sonication treatments. Both LC_S6 and LC_S8 differed significantly from the control (*p* < 0.05) but not significantly different from each other (*p* > 0.05). The partially active subpopulation Q2 (cF^+^ and PI^+^) did not increase after sonication (*p* > 0.05), which is consistent with the viability test results (Section 3.2). Finally, the non-metabolically active subpopulation Q3 (cF^−^ and PI^+^) increased significantly after sonication (*p* < 0.05), with no significant effect (*p* > 0.05) of treatment duration.

### 3.4 Confronting cultivable, viable, and metabolically active subpopulation

The absolute values of viable (AFU, SYTO^+^, and PI^−^), metabolically active (Q1, cF^+^, and PI^−^), and cultivable (cells grown on MRS agar) subpopulations for each sample were compared to estimate the overall response of the probiotic to sonication (Table 4). A paired Student's *t*-test was applied to evaluate the ultrasound treatment effects on the three parameters.

**Table 4:** Viable, metabolically active (Log/mL of events, mean values ± SD, *n* = 3) and cultivable subpopulations (Log CFU/mL, mean values ± SD, *n* = 3) of *Lacticaseibacillus casei* ATCC 393 before and after sonication.

**Sample**	**Subpopulations**		
	**Viable**	**Metabolic active**	**Cultivable**
LC_S0	8.88 ± 0.03^Aa^	8.96 ± 0.18^Aab^	9.16 ± 0.02^Ab^
LC_S6	8.60 ± 0.08^Ba^	8.58 ± 0.05^Ba^	8.42 ± 0.17^Ba^
LC_S8	7.21 ± 0.16^Ca^	8.37 ± 0.01^BCb^	6.37 ± 0.03^Cc^

LC_S0, non-sonicated, control; LC_S6, 6 min sonicated; LC_S8, 8 min sonicated.

Viable subpopulation: Active Fluorescent Unit (AFU), SYTO^+^ and PI^−^; Metabolically active subpopulation: Q1, cF^+^, and PI^−^; Cultivable subpopulation: cells grown on MRS agar.

Different capital letters in the same column and different lowercase letters in the same row indicate that the differences between the samples are significant (*P* < 0.05).

The viable subpopulation of *L. casei* ATCC 393 control culture was significantly (*p* < 0.05) lower than the subpopulation of cultivable cells, with 8.88 Log events/mL and 9.16 Log CFU/mL, respectively. However, no differences were found between the cultivable and metabolically active subpopulations or between the viable and metabolically active subpopulations (*p* > 0.05). On the other hand, no significant differences (*p* > 0.05) were observed among the three measured parameters after 6 min of sonication (LC_S6). Instead, a different pattern of results were obtained after 8 min of treatment. Data analysis revealed three distinct groups in the LC_S8 sample. The highest counts were observed for the metabolically active subpopulation (8.37 Log events/mL), followed by the viable subpopulation (7.21 Log events/mL) and, finally, the cultivable cells (6.37 Log CFU/mL).

The comparison between viable, cultivable, and metabolically active data revealed that osmotic stress reduced viability only in the control sample. In contrast, no further reduction in viability was observed in LC_S6. Moreover, viability, cultivability, and esterase activity of probiotics were similarly modulated by 6 min of sonication. However, the opposite phenomenon was observed after the more intense treatment. Notably, 8 min of sonication differentially modulated the three parameters in *L. casei* ATCC 393.

### 3.5 Growth recovery

**Figure 6:**
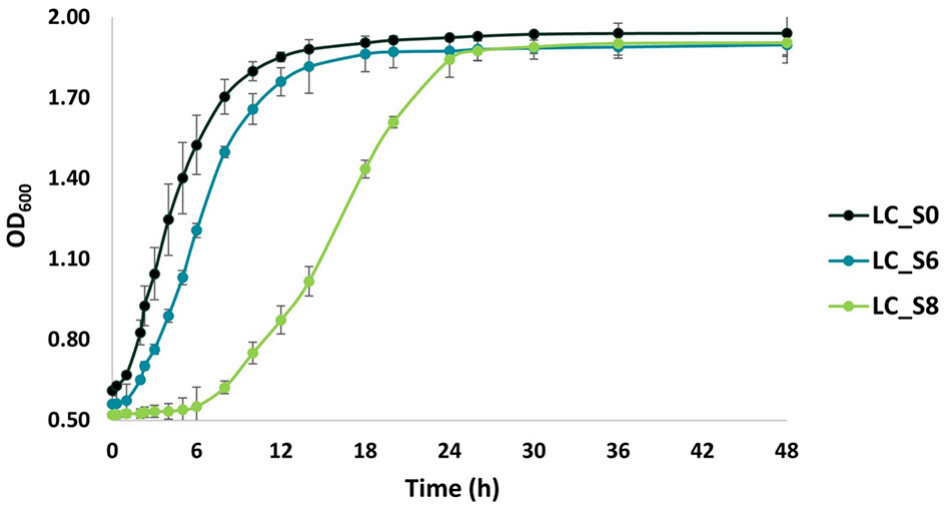
Growth curves of not sonicated and sonicated *Lacticaseibacillus casei* ATCC 393 over 48 h of incubation at 37°C in MRS broth. LC_S0: *L. casei* non-sonicated (control); LC_S6: *L. casei* 6 min sonicated; and LC_S8: *L. casei* 8 min sonicated. Data points represent the mean values ± standard deviation (SD, *n* = 3).

The ability of sonicated cells to restore growth was evaluated by cell density analysis (Figure 6). A two-way ANOVA analysis revealed three well-separated time intervals: 0–24, 24–36, and 36–48 h. Accordingly, all samples were significantly different within the first 24 h of incubation (*p* < 0.05). Instead, LC_S8 reached the same optical density (*p* > 0.05) as LC_S6 after 24 h of incubation. However, they were still significantly different from the control (*p* < 0.05). Finally, no significant difference (*p* > 0.05) was found among all samples after 36 h of incubation.

The growth rate (OD·h^−1^) is also reported in Table 5.

**Table 5:** Specific growth rate (μ_max_ mean values ± SD, *n* = 3) of *Lacticaseibacillus casei* ATCC 393 calculated at the early stationary phase.

**Sample**	**μ_max_ (OD·h^−1^)**
LC_S0	0.091 ± 0.02^a^
LC_S6	0.082 ± 0.01^b^
LC_S8	0.061 ± 0.02^c^

LC_S0, non-sonicated, control; LC_S6, 6 min sonicated; LC_S8, 8 min sonicated.

Different letters in the same column indicate that the differences between the samples are significant (*p* < 0.05).

The growth rate showed significant differences (*p* < 0.05) for all three samples. Moreover, the growth rate of the sonicated samples was significantly lower (*p* < 0.05) than that of the control sample, showing a strong growth delay.

## 4 Discussion

Modulation of probiotic metabolic pathways is of great interest for the development of functional foods with standardized and stable physicochemical and sensory properties. Attenuation is a technological approach intended to reduce undesirable changes that occur in food matrices, primarily due to sugar metabolism and matrix acidification.

Ultrasound has been previously described as a suitable tool to attenuate probiotic metabolism. It can directly alter the functionality of enzymes involved in sugar pathways or indirectly induce multiple damages, resulting in the perturbation of general metabolism. Independent of the type of action, a primary description of the ultrasound effects on a bacterial cell is given by its morphology. FCM revealed a reduction of the FSC and SSC after sonication. FSC depends on the cell's dimension; therefore, its decrease reflects the decrease in cell dimension and/or a decrease in chain length. Indeed, microscopic observation confirmed that the decrease in FSC is strictly correlated with the break of the chain between the cells, thus resulting in a single-cell morphology. Our results are similar to those of Peralta et al. (2022). The authors tested three ultrasonic treatments to evaluate the feasibility of ultrasound in accelerating the ripening phase in cheese production and avoiding the post-acidification defect. The scanning electron microscope (SEM) images of sonicated *Lacticaseibacillus paracasei* 90 revealed a change in the cellular morphology. Untreated lactobacilli exhibited a rod-shaped morphology, arranged in elongated rows. Instead, the sonicated lactobacilli were shorter with a reduced cell density. The ultrasound effect on cell morphology has been described by several authors (Liao et al., 2018; He et al., 2021). Even though the treatments were applied for different purposes and involved bacterial cells with varying morphologies, the studies reported similar effects. Specifically, shear forces and shock waves generated during the implosion of cavitation bubbles were found to impair the membrane structure and alter cell morphology.

Beyond morphological evaluation, FCM analysis combined with the use of fluorescent probes offers valuable insights into cellular changes that occur during various processes. Therefore, it was implemented to properly evaluate the effect of the ultrasound treatment on *L. casei* ATCC 393 cells. FCM data showed an intensity-dependent depletion of the viable subpopulation in both sonicated treatments. Moreover, ultrasound appears not to cause sublethal injuries. The damaged subpopulation of *L. casei* ATCC 393 did not increase after sonication, regardless of the treatment duration. The absence of sublethal damage in the subpopulation was also reported by Liao et al. (2018). The authors studied the ultrasound mode of action on *Escherichia coli* and *Staphylococcus aureus*, two model microorganisms. FCM detection revealed only green and red fluorescent emission, while double-positive red and green signals were not detected. This phenomenon is described as an “all-or-nothing” phenomenon (Clarke and Hill, 1970; Russell et al., 2000; Li et al., 2016). Moreover, the high amount of PI retention implies that the membrane of sonicated *L. casei* ATCC 393 is critically damaged and permeabilized. Li et al. (2016) found that, in Gram-positive bacteria, the cytoplasmic membrane is the primary target of ultrasound. Therefore, the response of cells to sonication, that is, the sublethal or lethal effect, depends on the membrane structure. Although the degree of permeabilization can reduce or improve the cultivability and metabolic activity of bacteria, it always leads to an increase in the PI-positive subpopulation (Li et al., 2016; He et al., 2021; Peralta et al., 2022). A review of existing literature reveals two contrasting effects of permeabilization on bacterial cells. On the one hand, low-level permeabilization (sublethal damage) has been found to have a positive impact on the cell, leading to enhanced activities such as fermentation, growth, and β-galactosidase activity (Shokri et al., 2020, 2021). On the other hand, high-level permeabilization (lethal damage) can significantly compromise cell viability (Zupanc et al., 2019). In our study, the membrane of *L. casei* ATCC 393 was impaired by the intense events generated during ultrasound propagation. Since attenuation aims to preserve cell viability, an improvement of the experimental design is needed to better control the loss of membrane integrity and thus cell vitality.

The complex phenomenon of cavitation triggers multiple reactions within a cell, leading to various injuries. Enhancement of comprehension of the ultrasound-induced metabolic alterations can be achieved by evaluating bacterial enzymatic activities. The current research aimed to study acidification as a specific enzymatic activity and esterase activity as a measure of general enzymatic activity, owing to their involvement in many cellular processes.

A strict relationship was observed between the time of exposure to ultrasound and the reduction of acidification abilities. The lower intensity treatment caused only a slight reduction in the acidification, while the higher intensity treatment caused a more effective modulation. A time-dependent effect was also previously observed by Racioppo et al. (2017) and Campaniello et al. (2020). Peralta et al. (2022) also found that ultrasound treatments had a strong and negative effect on the glycolytic enzymes in *L. paracasei* 90. In fact, when the ultrasound-treated probiotic was inoculated into milk, the decrease in lactose was significantly slower than that in the control. A high lactose content indicates lower utilization and, therefore, reduced enzymatic activity. These results highlight the technological impact of using ultrasound-attenuated probiotics for the probiotication of food matrices, where a low acidification capability is required.

cFDA and PI double staining first confirmed the results obtained by the double staining with SYTO 24™ and PI. The sublethally injured cells with residual metabolic activity (Q2) appear not to be affected by ultrasound treatments. Moreover, as previously observed by Li et al. (2016), the reduction in esterase activity is associated with an increase in cell permeability, independent of the treatment duration. However, the slight reduction of the esterase active subpopulation in both sonicated samples suggests that esterase can continue to carry out their activities, although the membrane is permeabilized (Jenkins et al., 2025).

The contrasting results of acidification and esterase activity highlighted the potential of using ultrasound to target multiple cell functions differentially.

Although the multifunctionality of ultrasound on different bacterial species is well established (Zupanc et al., 2019), the mechanism of action remains unknown. Subpopulation comparisons allow the discrimination of the ultrasound target on *L. casei* ATCC 393. We observed that, in LC_S6, the viability, cultivability, and esterase activity are modulated in a similar manner. Taking this into account, we speculate that the cell's primary exposure to osmotic stress activates stress response mechanisms that, through the phenomenon of cross-protection, could increase the cell's resistance to the non-specific stress (Gao et al., 2022). This hypothesis is supported by the results of Giordano et al. (2024). RNA sequencing provided insights into the complex transcriptional response of *L. casei* ATCC 393 after exposure to ultrasound. A higher number of differentially expressed genes (DEGs) was found in the sample sonicated for 6 min than in the sample sonicated for 8 min. These results revealed a robust defensive transcriptional response to multiple stresses. In addition, the overlap between the viable and metabolically active subpopulation also suggested that esterase remains active in permeabilized cells. Instead, the retention of viability and metabolic activity, along with the loss of cultivability, suggests the induction of the VBNC state in LC_S8 (Trinh and Lee, 2022). Our results are in agreement with those previously observed by Ananta et al. (2004). A treatment of 600 MPa on *Lacticaseibacillus rhamnosus* GG induced a reduction in cultivability but not in esterase enzyme functionality. Interesting findings have been reported by Ojha et al. (2018). Indeed, the authors studied the response of *Latilactobacillus sakei* DSM 15831 to several ultrasound frequencies by combining genomics and Phenotype Microarray (PM) technique. In particular, genome mapping with phenotypic variation for ultrasound-treated *L. sakei* revealed differences in nutrient utilization and certain metabolic pathways compared to the control sample. The pathways modified after ultrasound exposure were related to the utilization of carbon, nitrogen, phosphorus, and sulfur sources and depended on the ultrasound frequency. As a result, they obtained a high-throughput evaluation of metabolic activity. Therefore, the comparison of our data with the existing literature suggests that, although esterase enzymes may not be affected by ultrasound treatment, other metabolic pathways may be altered. It is, therefore, possible that esterase activity does not allow an adequate assessment of the ultrasound effect on metabolic activity.

Beyond the instantaneous ultrasound effect on cultivability and viability, it can also affect growth kinetics differently (Shokri et al., 2020; Speranza et al., 2020). Growth curve comparison revealed a time-dependent ultrasound effect on *L. casei* ATCC 393, with a considerable delay in growth observed in sonicated cells. Ojha et al. (2017) postulated that sonoporation—the formation of transient cavities or pores in the cell membrane—can occur in a controlled or random manner. Controlled sonoporation can have a positive effect on cell growth by forming a limited number of pores, thereby enhancing the nutrient uptake and the release of microbial end-products (Pitt and Ross, 2003). Indeed, random and higher levels of sonoporation lead to an uncontrolled efflux of cellular components and a delay in cell metabolism. However, our results on growth kinetics differ from those of Shokri et al. (2020). Using low-intensity ultrasound, they stimulated the growth and metabolic activity of *Lactococcus lactis* subsp. *lactis*, whereas we observed a growth delay in *L. casei* ATCC 393. However, the different shapes and sizes of cells in *Lc. lactis* subsp. *lactis*, and *L. casei* explain these contrasting effects. Cocci are more resistant to ultrasound waves than rods due to their lower cell surface-to-volume ratio (Bevilacqua et al., 2019). Consequently, since *L. casei* interacts with ultrasound by exposing a larger surface area, a higher number of cavities are formed.

Although the specificity of the obtained results represents the main limitation of the study, it also highlights the broad spectrum of action of ultrasound on bacteria and the need to develop a proper methodology for assessing the ultrasound effect on bacteria. Future investigations should focus on the application of metabolically attenuated probiotics in food matrices and on the expansion of probiotic species and strains by ultrasound treatment, which would enable a more comprehensive understanding of how ultrasound impacts different probiotics, ultimately supporting the development of more tailored and effective attenuation strategies for use in food processing. Finally, considering the purpose of using ultrasound as an attenuation strategy, it is necessary to review the ultrasound attenuation process and test further parameter combinations to prevent a decrease in membrane integrity and to achieve the desired metabolic modulation.

## 5 Conclusion

The ultrasound treatment induced a significant change in the cell structure, first observed by light microscopy and confirmed by variations in light scattering angles. The attenuation goal was achieved only under proper intensity conditions, and FCM data demonstrated that esterase activity was not impaired. These results underline that the metabolic functions of *L. casei* ATCC 393 are not modulated to the same degree when exposed to ultrasound waves, highlighting the need for specific metabolic studies. Additionally, the cultivability of the probiotic was negatively affected after sonication, resulting in reduced growth kinetics. However, the probiotic was able to restore its growth under suitable environmental conditions and time, highlighting the transient effects of ultrasound. Furthermore, the combined use of multiple FCM probes and data comparisons (plate count data vs. FCM data) allowed the identification and characterization of cell population heterogeneities, revealing the existence of an unculturable cell cluster in the sample sonicated for 8 min.

## Data Availability

The raw data supporting the conclusions of this article will be made available by the authors, without undue reservation.
